# Supplementary material for: Comparative multiomics analysis of cell physiological state after culture in a basket bioreactor
Source: Sci Rep. 2022 Nov 23;12:20161. doi: 10.1038/s41598-022-24687-4 (PMC9686226; doi:10.1038/s41598-022-24687-4)
Supplement: Supplementary file 1 — Supplementary Information 1. [file 41598_2022_24687_MOESM1_ESM.zip › raw data/Transcriptomic raw data/7.AS/2.dasplot/TE1vsTC1/A5SS/11_DQX1_NW_023666045.1_79418206_79418461_-@NW_023666045.1_79418673_79419246_-@NW_023666045.1_79418845_79419246_-.pdf]

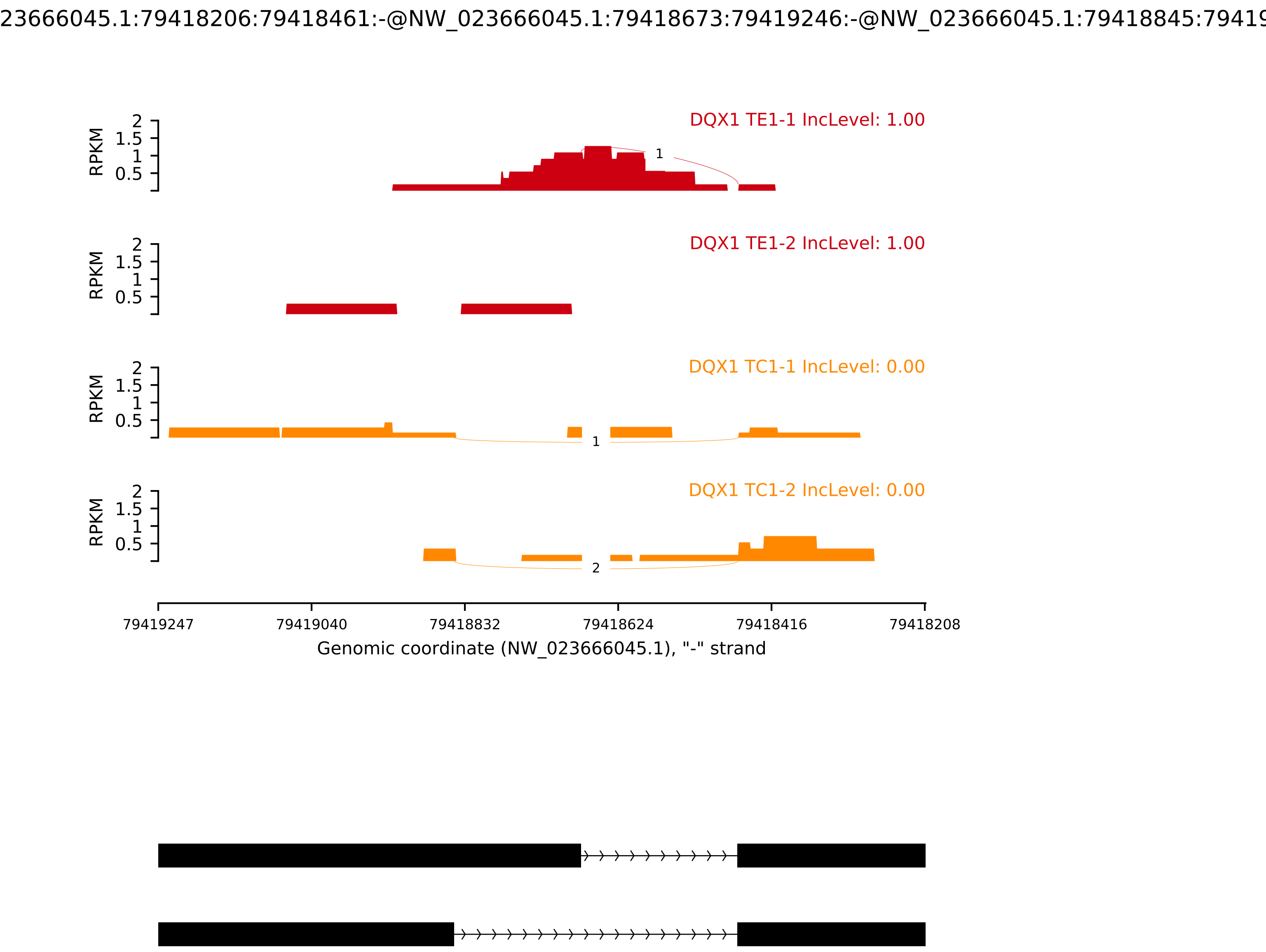
23666045.1:79418206:79418461:-@NW_023666045.1:79418673:79419246:-@NW_023666045.1:79418845:7941
DQX1 TE1-1 IncLevel: 1.00
DQX1 TE1-2 IncLevel: 1.00
DQX1 TC1-1 IncLevel: 0.00
DQX1 TC1-2 IncLevel: 0.00
RPKM
2
1.5
1
0.5
1
1
2
79419247
79419040
79418832
79418624
79418416
79418208
Genomic coordinate (NW_023666045.1), "-" strand
